# Most of our social scientists are not institution based… they are there for hire—Research consultancies and social science capacity for health research in East Africa

**DOI:** 10.1016/j.socscimed.2007.07.019

**Published:** 2008-01

**Authors:** Daniel Wight

**Affiliations:** Medical Research Council Social & Public Health Sciences Unit, University of Glasgow, 4 Lilybank Gardens, Glasgow G12 8RZ, UK

**Keywords:** Sub-Saharan Africa, Research capacity, Health social sciences, Research consultancies, Knowledge economy, East African universities

## Abstract

There is a serious shortage of senior African social scientists to lead health-related research in Africa. This is despite the existence of many African social science graduates, and decades of Northern funded research programmes intended to develop local capacity. To investigate the barriers to developing health social science research capacity in East Africa, 29 in-depth interviews, informal conversations and a group discussion were conducted with professionals in this field.

Respondents’ explanations for inadequate social science research capacity primarily related to under-development and global economic inequalities. However, a recurrent theme was the predominance of individually contracted research consultancies. These seem to divert university staff from academic research, supporting colleagues and training the next generation of researchers, stunt the institutional capacity of university departments, restrict the sharing of research findings and perpetuate donors’ control of the research agenda.

Although primarily due to macro-economic factors, limited research capacity in sub-Saharan Africa might be ameliorated by modifying the process by which much research is conducted. This exploratory study suggests that institutional research capacity might be strengthened if consultancy research were commissioned through institutions, rather than individuals, with the payment of substantial overheads.

## Introduction

There is a serious shortage of senior African social scientists to lead or manage health-related research in Africa (World Bank, 2000). This is despite the graduation of many African social scientists, and decades of Northern funded research programmes intended to develop local capacity (Nchinda, 2002; Simon, 2000). Whilst weak research capacity probably affects all areas of health, the HIV epidemic ‘offers a supreme test of how effectively African universities can respond to emerging challenges …’ (Zeleza, 2003, p. 84). Yet the shortage of senior social scientists is particularly apparent in sexual health. For instance, large-scale HIV/AIDS research programmes in both Tanzania and Uganda have been unable to recruit local social scientists to senior posts on international salaries, despite having trained local junior social scientists for over 10 years. Furthermore, international debates on social dimensions of sexual health, such as the hypothesis of permissive African sexuality (Caldwell, J, Caldwell, P, & Quiggin, 1989), are dominated by Northern academics, despite their sensitivity.

Health-related social science research capacity is the ability to investigate and define the social dimensions of health problems, set objectives, identify solutions and build sustainable institutions (cf. Sitthi-amorn & Somrongthong, 2000). Limited capacity is problematic at several levels. Most obviously, it requires non-local researchers generally unfamiliar with local life, reliant on interpreters, and prone to cultural misunderstandings with local fieldworkers. Service providers and policy makers have to base decisions on more superficial analyses, but ex-patriate-initiated research is less likely to have such practical application anyway (Costello & Zumla, 2000). At the broadest level, limited social science capacity restricts intellectual sovereignty (Zeleza, 2003), undermining political autonomy (RAWOO, 2002; Sitthi-amorn & Somrongthong, 2000).

The main explanations for limited research capacity have been identified in the literature as: inadequate resources for education at every level (Nchinda, 2002; Sall, 2003; Sitthi-amorn & Somrongthong, 2000); the drain of expertise to the North (Pang, Lansang, & Haines, 2002; Ramsay, 2002; Sall, 2003; Zeleza, 2003); dependence on Northern research funding (Jentsch & Pilley, 2003; Lansang & Dennis, 2004); inequitable access to the literature (Lansang & Dennis, 2004); unbalanced North–South research collaborations (Costello & Zumla, 2000; Jentsch & Pilley, 2003) and poor support from government (Nchinda, 2002; Sall, 2003; Sitthi-amorn & Somrongthong, 2000). Some see the perpetuation of inadequate research capacity as replicating the imbalance in global trade relationships (Zeleza, 2003) and essentially semi-colonial (e.g., Costello & Zumla, 2000). Others assume the good intentions of funders and research partners, but identify the perverse consequences of North–South collaborations (Edejer, 1999), such as poaching senior researchers from local institutions. Either way, limited research capacity in Africa should be an ethical issue for Northern researchers working there.

Whilst the main causes of weak research capacity are clearly macro-economic, the *processes* of conducting research might contribute to the problem and may be more readily modified (Green, 2003). Some reports have suggested that research consultancies, whilst augmenting meagre incomes, might marginalise teaching and research and undermine social science scholarship (Kwesiga, Mbago, & Chimanikire, 2000; Menken, Blanc, & Lloyd, 2002; Mkandawire, 1998; Sall, 2003). Being highly prescribed, consultancies have also been said to exacerbate the way African social science research is narrowly policy-bound (Allen, 1986; Sall, 2003), and increase Northern dominance of the research agenda (Mkandawire, 1998). ‘…consultants do not frequently choose to contradict the donor's agenda, … this would decrease their chances of … consultancies in the future.’ (Rossi, 2004, p. 27). Consultancies have been described as particularly problematic in East Africa (Sall, 2003), the University of Nairobi being called a ‘consultancy university’ (Allen, 1986, p. 25).

There have been many attempts to strengthen research capacity in developing countries, leading sponsors being the WHO and other UN agencies (e.g., the Special Programme for Research and Training in Tropical Diseases (TDR); UNDP/World Bank/WHO, 2003), national development agencies (e.g., Swiss, Canadian and Japanese), foundations such as Rockefeller and NGOs such as the Population Council. However, there is little evidence about the most effective approach (Simon, 2000), and debates continue over, for instance, investing in individuals or institutions (Costello & Zumla, 2000; Nchinda, 2002), whether post-graduate training in the North exacerbates the brain drain (Nchinda, 2002), and Southern control of research budgets (Lansang & Dennis, 2004; Nchinda, 2002). The role of consultancies, however, has received scant attention and almost exclusively in the grey literature.

In order to investigate the poor capacity for health-related social science research in East Africa, the processes perpetuating it and possible ways to improve it, a small-scale exploratory study was conducted in Tanzania, Kenya and Uganda. The general findings have been reported elsewhere (Wight, 2005). This short report focuses on the individualised nature of research activity and the role of individual research consultancies in shaping research capacity.

## Methods

In 2003 and 2004, I conducted in-depth interviews with 29 leading professionals conducting, commissioning or supporting health-related social science research in East Africa (Table 1). The findings are biased towards Uganda, 18 interviewees being Ugandan, four Kenyan, three British, two North American, one Tanzanian and one Nigerian. This was primarily a snowball sample including seven senior social scientists from Makerere, the oldest university in East Africa with by far the largest research function in Uganda, and others from the London School of Hygiene and Tropical Medicine (three), the Universities of Nairobi (two) and Dar es Salaam (one) and from the leading independent research centres and research-supporting NGOs in Uganda and Kenya. None of the new universities were represented. To protect respondents’ anonymity small institutions are not named.

**Table 1:** Sample of interviewees

No.	Post	African	British or North American
2	Directors of research programmes	1m	1m
6	Heads of university departments	4m, 2f	
5	Senior university researchers	1m, 2f	1m, 1f
2	Non-senior university researchers	1m, 1f	
1	Director of independent research centre	1m	
6	Research staff in independent research centres	3m, 2f	1m
2	Directors of NGOs facilitating research	1m	1m
1	Junior staff in NGO facilitating research	1f	
4	Senior staff in health-related government departments	3m, 1f	
29	Total	15m, 9f	4m, 1f

m, male; f, female.

The interview schedule covered leadership of local social science research, training and career paths, ways of strengthening research capacity and barriers to this. The full schedule is available (as Appendix A).

Informal conversations were held with five senior and one junior researcher from the University of Dar es Salaam and National Institute for Medical Research, Tanzania, and nine junior researchers from Makerere and the MRC Programme on AIDS in Uganda. A group discussion was held with four of the Ugandans (three men, one woman), following the same schedule used for the in-depth interviews.

The interviews were summarised according to analytical themes. There was considerable concordance in accounts, which were not patterned by gender. Divergent opinions are considered in the Discussion. Interviewees were circulated the main report for comments and to confirm that their views were presented accurately. Four provided comments.

## Findings

### Severity of the problem

Nearly all those interviewed thought that there is a serious shortage of social science research capacity in East Africa, the few really good social scientists being overworked and overwhelmed with requests for collaboration. Most academic health-related social science research in Uganda was said to be run by Northerners, yet Uganda was thought to have stronger capacity than Kenya, with Tanzania coming third. Particular limitations identified were in qualitative research, analysis and writing skills, and health-related specialisms.

Interviewees stated that the vast bulk of social science research in East Africa is commissioned by NGOs or government departments, mostly funded from the North; consequently, it is highly applied and determined by external priorities. The few opportunities for academic research were said to come primarily from Northern researchers who win the funding, resulting in unbalanced collaborations.

### Research processes: inter-collegiate support

Poor social science capacity was primarily related to under-development and global economic inequalities: very poor schooling, talented students choosing high status vocational courses, poor university facilities and teaching, research funded through Northern institutions, and the drain of senior researchers abroad (Wight, 2005). The problem has been exacerbated by the death of many junior and mid-level Researchers from AIDS (Pfau & Barton, 2004, RAWOO, 2002, Zeleza, 2003). However, the individualised nature of departments and lack of collegiate support were also identified as unhelpful. This was primarily attributed to lack of resources and staff's reliance on individual research consultancies, resulting in no writing skills training and limited publishing experience to share. A head of department observed that senior staff rarely co-author papers with junior colleagues, due to ‘the culture of individualism.’ In her department there was no formal system to support junior researchers, though she was planning a mentoring system. Staff rarely seem to comment on their colleagues’ draft papers; one senior respondent estimated that at Makerere only 1% of colleagues would have time.

### Research processes: consultancies

Most of the research work conducted by social scientists in East Africa is in the form of consultancies. The proportion of academics’ time spent on them is unclear (Kwesiga et al., 2000), partly perhaps to disguise this from supervisors, but most estimates were around 50% of working time. Teaching takes up much of the rest, with very little for academic research.

… in Makerere you can spend your entire time just working on very well paid, short-term consultancy studies for NGOs, … who want something done in three weeks, and will pay you very well … (Senior researcher, previously Uganda)

Extremely low university salaries create a powerful incentive for consultancies. A research associate's salary might be $250/month, while consultancies can pay $100–$250/day. In one research institute consultancies augment salaries from around $400/month to about $5000. A head of department explained: ‘… to rely on your salary would never make ends meet at all.’ Furthermore, in contrast to regular salaries, most researchers can avoid declaring consultancy fees for tax (30%).

Research commissioners, predominantly government departments or NGOs, usually seek a contract with individuals, or sometimes consultancy firms, but rarely with university departments. Private consultancy firms, often constituted for a particular brief, usually employ university staff to help with the bid and subsequent research.

Commissioning bodies are reportedly unwilling to pay overheads to institutions, and when they do, they are generally very low, e.g., 5–20% in Makerere departments, 20% at the University of Dar es Salaam, and a maximum of 15% at a Kampala independent research centre. The senior management at Makerere were said to encourage departments to become consulting firms and demand 30% overheads, but this leads university staff to work independently, undercutting university departments and earning more.

The predominance of research consultancies is critical for the development of research capacity. Financial insecurity leads researchers to take on any work available, and consequently:

There are no research traditions being developed …. We are social scientists but very few are specialists … (Faculty dean)

Consultancy work also inevitably restricts academics’ time for teaching and supervision. At Dar es Salaam and Makerere consultancies should not interfere with normal academic work, but this is difficult to enforce:

He will leave you. And who loses? This is the person you have trained up to PhD level, and now he is leaving you, and you have no one to teach … (Head of department)

Writing consultancy reports provides little incentive to develop analytical skills. Reports generally involve very tight timetables with little opportunity for peers’ critical input, are descriptive and have limited dissemination (sometimes for internal use only). Several researchers said they do not publish from consultancies because they need the funder's permission, but none knew of it being refused. More plausibly, there is rarely time for such writing. Consequently, the CVs of highly experienced researchers often list numerous consultancy reports but very few journal publications, jeopardising their applications for senior jobs.

The conflict between consultancies and academic publications reportedly generates a professional culture in which: ‘the point is to try and chase the quick money, and not take advantage of the chance of academic growth … people don’t value it very much.’ A faculty dean commented:

Consultancies is not building the capacity of the person who is doing it. …. [Some] have even refused scholarships to do PhDs because they were busy doing consultancies.

Only two interviewees questioned the inevitability that consultancies detract from publications or teaching: ‘… consultancies can … be a source of writing … [and] training.’ (Director large research programme).

The high remuneration from consultancies, and tensions with teaching responsibilities, might encourage researchers to become full-time consultants. However, few do this because commissioners seek the ‘recognition and visibility’ of ‘high powered people’ in established university posts. Furthermore, it would be too insecure: ‘You would earn *much* more in the short-term, but then you would be unemployed in the long-term.’

### Strengthening research capacity and likely barriers

Respondents proposed many ideas to strengthen health-related social science research capacity (see Wight, 2005). Here I focus on those to modify the individualism of research practice and consultancies.

Five senior interviewees identified the need to develop writing skills, for instance ‘to guide you through … the very complicated processes … and requirements’ to publish in international journals. Suggestions included experienced and in-experienced staff co-authoring, mentoring systems and support networks. The director of a research-facilitating NGO advocated posts dedicated to writing support, but with salaries adequate to prevent appointees taking on consultancies.

The potential advantages of institutional research consultancies were explored, and in particular establishing a norm of significant overheads, e.g., 30%. Everyone approved in principle. Overheads could be used for: libraries, computing and internet access; department-initiated research; disseminating reports; training staff and developing writing skills. Institutional consultancies might facilitate a more collective approach to research and assist management by departmental heads. Furthermore, paying overheads might benefit commissioners since they could require reports to be published, at least in an on-line journal.

However, several objections to institutional consultancies were also raised.

The culture of institutionalising things is not there. Many think the institution is a barrier to them. And … the bureaucracy, you know, many people would prefer to have the money in their own accounts …. (Head of department)

Researchers anticipated the frustrations of inefficient institutional administrations, with long delays in finalising contracts or being paid. It was feared that, since some universities do not allow departmental bank accounts, the central administration might appropriate funds raised through departmental consultancies. Furthermore, fees would not only have to be shared with the institution, but would have to be declared for tax. Consequently, the director of a large programme thought: ‘… people will just try to get around it. They will get consultancies privately.’

Several interviewees said that commissioning agencies would not ‘… want to pay the institutional fee.’

All the American universities [have] institutional overheads, but tell DfID that [they] have to be factored in [in Kampala] …: “Oh, no!” …. How am I supposed to run the project without institutional overheads? … they have the mentality that they can do it on the cheap. Africa is poor, but it is not cheap! (Director of large programme)

It was also argued that some agencies want to commission specific individual researchers, and that individual consultancies incentivise good work produced on time.

Some large donors practice their policies of strengthening institutional capacity by only contracting research through institutions, e.g., the Carnegie and Rockefeller Foundations, the World Bank, and the Swedish and Norwegian development agencies. However, some interviewees thought a concordat with all commissioning agencies to pay minimum overheads would be unrealistic, since East Africa is too dependent on donors.

## Discussion

Although most respondents were Ugandan, the data from Kenya and Tanzania suggest that these findings apply across East Africa, while the broader literature (e.g., Carlsson & Wohlgemuth, 1996; Sall, 2003; Zeleza, 2003) and contacts with researchers elsewhere suggest they are relevant to much of sub-Saharan Africa. There is a serious shortage of health-related social science research capacity in this region, as evidenced by the Northern intellectual leadership of most academic research. This perpetuates ‘the international intellectual division of labour whereby African … social scientists … import appropriate … theory and, at best, export empirical data.’ (Zeleza, 2003, p. 111) African countries’ limited ability to define for themselves their problems and the solutions may have very practical consequences. For instance, Cleland and Watkins (2006, p. 2) argue that Africans’ frustratingly slow response to the HIV epidemic is because ‘the problem, and the remedies, were socially constructed in the West ….’

Like most previous studies, this one points to global economic inequalities as the primary cause of limited research capacity. However, unremarked in nearly all the published literature, these findings also suggest that the problem is perpetuated by the highly individualised character of research in East Africa, fuelled by the dominance of individually-contracted consultancies. ‘Most of our social scientists are not institution based, whether NGO or private. They are there for hire.’ (Faculty dean) Such consultancies seem to stunt research capacity: reports are generally not disseminated, thus not contributing to collective understandings, university departments are denied overheads, and staff are diverted from teaching, supporting colleagues, or publishing. Furthermore, consultancies exacerbate the narrow policy orientation of African social science research (Allen, 1986; Rossi, 2004; Sall, 2003).

Given their prominence, this study explored the potential for research consultancies to be used to strengthen research capacity, which to date has been largely ignored in the wider literature. The *principle* that consultancies should be contracted with institutions, rather than individuals, with overheads of around 30%, was widely accepted. This could fund many initiatives to strengthen research capacity, facilitate a more collective approach to research, and in the longer term might mean commissioning agencies get better value. Some African universities and research centres already regulate the division of consultancy fees between researchers and their institution, e.g., the University of KwaZulu-Natal and the REACH Trust, Malawi (Theobald & Nhlema, in press). However, established individual consultants would probably oppose institutionalisation, given very low university salaries and lack of confidence in departmental administration, due to experience of patronage, mismanagement and corruption (Zeleza, 2003). Furthermore, commissioning agencies were said to prefer individual consultancies as cheaper and more straightforward.

By and large, initiatives to strengthen research capacity do not address the issue of research consultancies, although in practice they are in competition for researchers’ commitment. This was clear in an academic research centre sponsored by an international NGO where researchers are prohibited from consultancy work. However, as noted above, some large donors further the development of institutional capacity by only contracting research through institutions, not individuals.

Unsurprisingly, respondents’ accounts were shaped by their professional and institutional positions, for instance leading them to defend their staff or externalise problems (see Wight, 2005). African interviewees gave more emphasis to economic factors, research commissioners’ restrictions and exclusion from Northern-dominated academic networks, while Northern interviewees were more likely to contrast East African with Northern professional cultures. However, these cultural differences were usually attributed to underlying structural/economic factors. Only one interviewee, an African, explicitly attributed inadequate research capacity to a global economy of academic research, in which Northern institutions actively maintain their dominance. Notably, the most critical reports of East African research came through informal conversations, rather than recorded interviews.

This has only been an exploratory study. Further research needs to clarify: the scale of individual consultancies across East Africa; whether revising commissioning practices would seriously contribute to research capacity; and, hardly represented here, the views of agencies commissioning consultancies.

While the underlying causes of poor research capacity require global economic reform, this study also points to the importance of individually contracted research consultancies in perpetuating the problem. Although they greatly augment meagre university salaries, they also seem to divert university staff from academic research and training the next generation of researchers, stunt the institutional capacity of university departments, restrict the sharing of research findings and perpetuate donors’ control of the research agenda. Commissioning bodies committed to strengthening research capacity should consider devising research contracts, and means to improve university administration, that ameliorate rather than exacerbate the problem.

## Appendix A Supplementary materilas

Online supplimentary materials
