# Supplementary material for: Most of our social scientists are not institution based… they are there for hire—Research consultancies and social science capacity for health research in East Africa
Source: Soc Sci Med. 2008 Jan;66(1):110–6. doi: 10.1016/j.socscimed.2007.07.019 (PMC2430159; doi:10.1016/j.socscimed.2007.07.019)
Supplement: Supplementary file 1 — Online supplimentary materials [file mmc1.pdf]

APPENDIX

Generic interview schedule for in-depth interviews

1. Is there a problem [in your view] with local participation in social science research?

Which kinds of social science research in Uganda are led by local researchers?
- e.g. by sector: government research, NGO research, academic research
- by subject area: demography, marketing, education, health

To what extent is social science sexual health research in Uganda led by local researchers?
- what proportion of major research projects are initiated, managed and written-up by Ugandans?
- what level of research do Ugandans work at?
  e.g. grant holders (PIs); senior research managers; graduate researchers; non-graduate researchers

2. Where do social scientists come from? [supply issues]

How many social scientists trained in this country?
- graduates
- post graduates: MAs/MSc or PhD

Which universities/ colleges train social scientists?

3. Where do social science graduates go? Competition for social scientists. [demand issues] Is there an issue with recruitment?

Where do trained social scientists end up working?

What salary structure is there in your research programme/ institution?
- local and international salaries: rationale for distinction

Where are jobs advertised?
- are vacancies circulated formally and/or informally? What networks?

To what extent do post-doctoral researchers devote time to consultancy research?
- what is the output from these consultancies?
- what quality is it?
- how does this affect acquisition of skills to write successful research proposals and write for publication in academic journals?

Are some jobs perceived to be for ex-pats?
- what links are there with Makerere?
- are there any pro-active recruitment practices?

Might there be a lack of confidence amongst local social scientists? (Cultural dependency?)

4. Issues of education and training. Academic practices, medium of communication, resources. Motivation to develop skills.

What is the quality of social science courses?
- do staff have to rely on consultancies for income?
- does reliance on consultancies affect teaching staff's teaching quality?

What styles of teaching? Discursive/ didactic?

What language is used in secondary school and university?
- how does this help or handicap students?
- access to literature?
- implications for writing for publication

What are the main areas in which training is needed?
- do many social scientists have experience of writing?
- does assessment of researchers/ research institutions allow much time to be devoted to developing local researchers' writing skills, when quicker for ex-pats to write papers?

What are the pros and cons of local researchers doing post-grad. degrees in West?
e.g. value of international exposure
- value of international ideas/ theory
- are degrees always appropriate
- does it encourage researchers to stay in developed countries
- does it perpetuate expertise and resources in the West

How do you motivate staff to develop their skills?
- is there any motivation to get publications?

What is the usual system for allocation of authorship for journal articles?
- how widespread is practice of 'gift' authorship?

5. How are 'social scientists' perceived in Uganda? Is there a professional grouping? If so, what does it do? What is social status of social scientists? Is it seen as a worthwhile career? [retention issues]

Is there any professional identity as a researcher, a social scientist or a scientist in Uganda?

What kind of professional organisations or networks are there for social scientists?

What is status of social scientists compared with other professions?

What career structure is there?

Could large scale research programmes funded by foreign donors do more to provide career progression for researchers?

6. How might things be improved? Have you tried anything, different strategies? Do you think it matters?

Can you suggest any other possible solutions to help develop social science research capacity?

Would it be helpful if consultancies were contracted with university departments or research centres, rather than individuals, and it became established practice for institutions to require significant overheads, e.g. 30%?

- how could these overheads be usefully used?

Do you think there are biases between local and Western researchers in access to research funds?

- how do these operate?

Do you think there are biases between local and Western researchers in the way academic journals review and accept papers for publication?

- how do these operate?
